# Comparison of Female Ovarian Reserve Before vs After COVID-19 Vaccination

**DOI:** 10.1001/jamanetworkopen.2023.18804

**Published:** 2023-06-16

**Authors:** Liubin Yang, Samantha Neal, Tiffany Lee, Andrew Chou, Amy K. Schutt, William Gibbons

**Affiliations:** 1 Division of Reproductive Endocrinology and Infertility, Department of Obstetrics and Gynecology, Baylor College of Medicine, Houston, Texas; 2 Center for Innovations in Quality, Effectiveness, and Safety, Michael E. DeBakey Veteran Affairs Medical Center, Houston, Texas; 3 Section of Infectious Diseases, Department of Medicine, Baylor College of Medicine, Houston, Texas; 4 Texas Fertility Center, Austin, Texas

## Abstract

This cohort study examines the association of COVID-19 vaccination with levels of anti-Mullerian hormone and antral follicle count in women seeking fertility treatment.

## Introduction

Understanding the effects of the COVID-19 vaccine remains a public health priority, including how the vaccine affects female ovarian reserve. The American Society for Reproductive Medicine recommends using anti-Mullerian hormone (AMH) levels and sonographic antral follicle count (AFC) in combination to assess ovarian reserve.^1^ Anti-Mullerian hormone is a glycoprotein produced by the granulosa cells of growing follicles in the ovaries and correlates with the functional ovarian pool.^2^ Antral follicle count is a sonographic measurement of the bilateral ovaries during the follicular phase of the menstrual cycle.^1^ Previous work^3,4,5^ demonstrated no changes in AMH levels following vaccination. Given the variations in AMH assays and the need for cross-referencing other biomarkers, we studied the association of COVID-19 vaccination with AMH level and AFC among women seeking fertility treatment.

## Methods

We conducted a retrospective cohort study of new female patients seen from January 4, 2016, to December 16, 2021, at the Texas Children’s Family Fertility Center in Houston. This study was approved by the Institutional Review Board at Baylor College of Medicine, and a formal ethics committee waiver of consent was approved due to use of deidentified data. This study followed the STROBE reporting guideline. The primary outcome was ovarian reserve as defined by AFC or AMH level. Heteroskedastic data were corrected using heteroskedastic linear regression with outcome variables age, body mass index (BMI; calculated as weight in kilograms divided by height in meters squared), hemoglobin A_1c_ (HbA_1c_) level, type of AMH assay (former, current, or transition), and vaccination status (prevaccination or postvaccination). The dependent or outcome variable was AMH level or AFC. Missing data entries were excluded from the study. Statistical tests were performed using Stata/IC, version 15.1 (StataCorp LLC). Two-sided *P* < .05 indicated statistical significance.

## Results

Of the 1655 patients evaluated, 974 met criteria for AMH (mean [SD] age, 34 [4.7] years) and 1222 for AFC (mean [SD] age, 34 [4.7] years) analyses (eFigure in Supplement 1). Data on race and ethnicity for individual patients were not collected as they were not part of the initial study design. The AMH analyses included 836 patients in the prevaccination and 138 in the postvaccination groups (87 BNT162b2 [Pfizer-BioNTech], 28 mRNA-1273 [Moderna], 9 Ad26.COV2.S [Janssen], and 14 unknown). The prevaccination group was younger and had a lower mean AMH level, but no differences in median BMI and HbA_1c_ level (Table 1). The mean (SD) number of doses was 2.19 (0.68), and postvaccination AMH level was measured between 4 and 356 days after the first vaccine dose. Thirty-seven patients had documented natural immunity to COVID-19 but were excluded due to missing demographic information. There was a significant difference among vaccinated patients relative to the type of AMH assay (259 [31%] in the prevaccination vs 137 [99%] in the postvaccination groups with current assay; *P* < .001). In a linear regression model (Wald χ^2^_6_ = 192.63; *P* = .006), only age (95% CI, 0.004-0.042) and AMH platform (former assay, 95% CI, 0.084-0.737) but not vaccination status (95% CI, −0.054 to 0.536) were associated with AMH value (Table 2). Within this cohort, 70 patients demonstrated no change in mean (SD) AMH levels measured before (3.83 [4.56] ng/mL) and after (3.86 [4.31] ng/mL) COVID-19 vaccination (95% CI, 0.491-0.566; *P* = .89). The median AFC was 18 (IQR, 11-28) for the prevaccination group and 20 (IQR, 12-29) for the postvaccination group. In a linear regression model (χ^2^_4_ = 234; *P* = .004), age (coefficient, −0.060 [95% CI, 0.0004-0.036]) and logBMI (coefficient, −0.050 [95% CI, 0.222-0.915]), but not vaccination status (coefficient, 0.068 [95% CI, –0.322 to 0.184]), correlated with total AFC. In a subgroup analysis of the postvaccination population adjusted for age, BMI, and HbA_1c_ level, positive vaccination status was associated with higher AMH level (median, 3.3 [IQR 1.3-5.5] vs 4.2 [IQR, 1.5-8.8] ng/mL; 95% CI, 0.502-2.301 ng/mL).

**Table 1.** Baseline Demographic Values Between the Prevaccination and Postvaccination Groups

Characteristic	Patient group		*P* value^a^
	Prevaccination	Postvaccination	
AMH study			
No. of patients	836	138	NA
Age, mean (SD), y	34.2 (4.7)	35.1 (4.6)	.07
BMI, median (IQR)	26.2 (23.0-32.0)	25.1 (22.0-30.2)	.06
HbA_1c_ level, median (IQR), %	5.1 (4.9-5.4)	5.1 (4.9-5.3)	.28
AMH level, median (IQR), ng/mL	2.6 (1.2-5.0)	4.2 (1.6-8.8)	.001
AFC study			
No. of patients	1081	141	NA
Age, mean (SD), y	34.1 (4.6)	35.0 (5.1)	.03
BMI, median (IQR)	25.9 (23.0-32.0)	26.3 (23.0-32.0)	.99
HbA_1c_ level, median (IQR), %	5.1 (4.9-5.4)	5.1 (4.9-5.3)	.21
AFC, median (IQR)	18 (11-28)	20 (12-29)	.49

Abbreviations: AFC, antral follicle count; AMH, anti-Mullerian hormone; BMI, body mass index (calculated as weight in kilograms divided by height in meters squared); HbA_1c_, hemoglobin A_1c_; NA, not applicable.

^a^ Calculated using an unpaired 2-tailed *t* test for age and Kruskal-Wallis analyses for all other comparisons.

**Table 2.** Statistical Estimates of Variable Coefficients in Linear Regression Models

Vaccination status associated with outcome	Crude coefficient (95% CI)	Adjusted coefficient (95% CI)
AMH	1.777 (0.979 to 2.554)	NA
AMH covariates^a^	0.250 (0.012 to 0.488)	0.241 (−0.054 to 0.536)
AMH new assay^b^	1.276 (0.323 to 2.229)	1.401 (0.502 to 2.301)
AFC	0.035 (−2.175 to 2.245)	−0.069 (−0.322 to 0.184)

Abbreviations: AFC, antral follicle count; AMH, anti-Mullerian hormone.

^a^ Refers to regression data with addition of other variables (age, body mass index, and hemoglobin A_1c_ level) with (adjusted coefficient) and without (crude coefficient) heteroskedastic correction.

^b^ Represents subgroup analysis of patients who had AMH values obtained from the new AMH assay on or after 2020.

## Discussion

These findings suggest that COVID-19 vaccination is not associated with changes in ovarian reserve by multiple biomarker assays of AMH and AFC, supporting previous studies on AMH.^3,4,5^ The AMH difference was not clinically significant on subgroup analysis. Study limitations include the small number of patients who received vaccination, factors associated with ovarian reserve that were not included in the study, and survival bias, as only patients who pursued fertility treatment were included. Outcomes among those who did not pursue treatment were not captured.

## Notes

Supplement 1. **eMethods.** Study Design and Population and Analysis **eFigure.** Flowchart of Selection of Subjects for AMH and AFC Analysis

Supplement 2.
Data Sharing Statement
