# Supplementary material for: Comparison of Female Ovarian Reserve Before vs After COVID-19 Vaccination
Source: JAMA Netw Open. 2023 Jun 16;6(6):e2318804. doi: 10.1001/jamanetworkopen.2023.18804 (PMC10276301; doi:10.1001/jamanetworkopen.2023.18804)
Supplement: Supplement 1. — eMethods. Study Design and Population and Analysis eFigure. Flowchart of Selection of Subjects for AMH and AFC Analysis [file jamanetwopen-e2318804-s001.pdf]

Supplementary Online Content

Yang L, Neal S, Lee T, Chou A, Schutt AK, Gibbons W. Comparison of female ovarian reserve before vs after COVID-19 vaccination. *JAMA Netw Open*. 2023;6(6):e2318804. doi:10.1001/jamanetworkopen.2023.18804

eMethods. Study Design and Population and Analysis
eFigure. Flowchart of Selection of Subjects for AMH and AFC Analysis

This supplementary material has been provided by the authors to give readers additional information about their work.

© 2023 Yang L et al. *JAMA Network Open*.

eMethods. Study Design and Population and Analysis

Study design and population

We conducted a retrospective cohort study of women at the Texas Children's Family Fertility Center in Houston, Texas. This study was approved by the Institutional Review Board at Baylor College of Medicine. A waiver of consent was granted by the IRB committee. All the participants were new patients seen from January 4, 2016, to December 16, 2021. Data collection was conducted from November 2021 to May 2022. Inclusion criteria were new patient visits, documentation of vaccination in the electronic health record, presence of anti-Mullerian hormone (AMH) laboratory value in ng/mL, and presence of ovarian antral follicle count (AFC) by ultrasound. Exclusion criteria were incomplete data and no documentation of vaccination after December 2020. The vaccination date was documented in the electronic health record as either a medication administration visit or a historical documentation in Immunization Record section. We compared the date of the AMH or AFC testing with the vaccination date. For those who had the ovarian reserve testing prior to vaccination, we designated them to be the "pre-vaccination group".

Demographics including age, body mass index, laboratory values including hemoglobin A1c were also obtained. Primary outcome was ovarian reserve as defined by the value of antral follicle count on ultrasound or anti-Mullerian hormone value. Antral follicles were manually counted on each ovary by transvaginal ultrasound by a reproductive endocrinology and infertility physician at the first patient visit. Follicles measured were

© 2023 Yang L et al. *JAMA Network Open*.

between 2 and 9mm in diameter. Counts were also verified by a second physician. Total antral follicle count is the summation between the counts from the left and right ovaries.

A clinically relevant difference in AMH includes value <1ng/mL indicative of diminished ovarian reserve or >5ng/mL indicative of high ovarian reserve associated with PCOS. Values in between are predictive of average response to ovarian stimulation as clinically gauged. Therefore, a difference of 1ng/mL is not clinically significant. Similarly, AFC values <5 indicate poor ovarian reserve and values >20 indicate high ovarian reserve. There is month to month variation of +/-4 for AFC.

AMH assay

During the collection period, the laboratory assay platform for AMH changed. Prior to 2019, the group used “former assay” to designate the prior assay being used. During 2019, a “transition assay” period was designed for some assays that were conducted using the former type and some were done using the new assay. From 2020 and onwards, all patients underwent “current assay” for AMH analysis.

Bias

Potential confounders were demographics variables collected including age. To minimize performance and detection biases, the personnel determining antral follicle counts were blinded to the data collection and analysis. The personnel collecting data including laboratory values were blinded from data analysis and outcome measurements.

© 2023 Yang L et al. *JAMA Network Open*.

Sample size

In order to detect a 10% difference in antral follicle count (20 vs 18) with a standard deviation of 10, enrollment ratio of 3:1 (pre-vaccination:post-vaccination), alpha of 0.05 and a power of 80%, we estimated total sample size of 1,048 with cohort ratios of 262 to 786.

Statistical analysis

Normality of data was verified with Shapiro-Wilk tests. Chi-square, Kruskal-Wallis and student’s t-test were used to calculate differences in baseline characteristics. Data that was not normally distributed underwent log transformation. Variance of the residuals was tested using Breusch-Pagan/Cook-Weisberg test for heteroskedasticity. Heteroskedastic data was corrected using heteroskedastic linear regression. The predictor variables were age (years), body mass index (BMI, the weight in kilograms divided by square of the height in meters), percent hemoglobin A1c (HbA1c), type of AMH assay (former, current, transition), and vaccination status (pre or post-vaccination). The dependent or outcome variable was anti-Mullerian hormone (AMH, nanograms per milliliter) or antral follicle count (AFC). Missing data entries were excluded from the study. Statistical tests and graphs were performed using Stata/IC15.1.

© 2023 Yang L et al. *JAMA Network Open*.

eFigure. Flowchart of Selection of Subjects for AMH and AFC Analysis

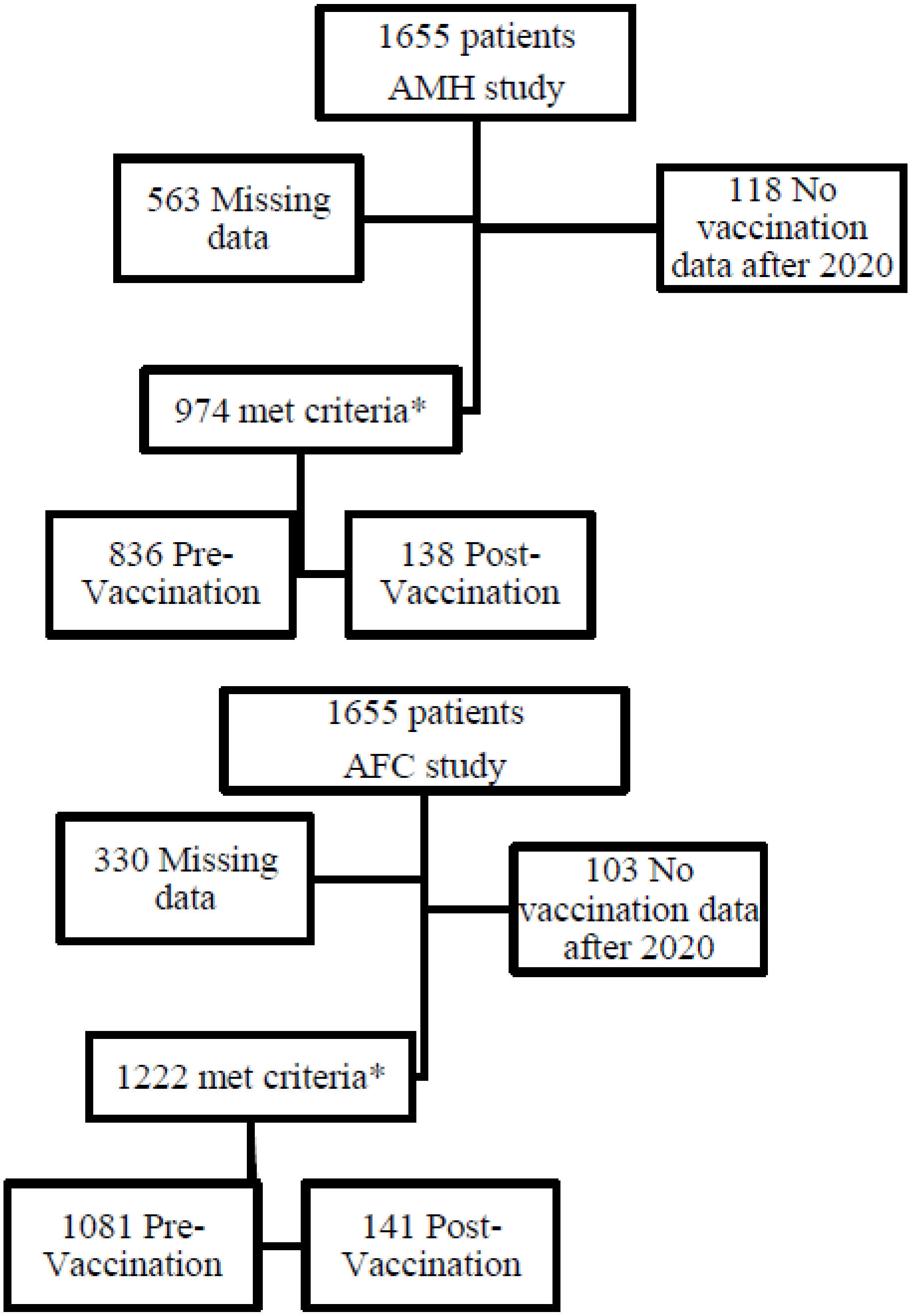

*Exclusion criteria: no BMI, no HbA1c, no AMH, unknown vaccination status after Dec 2020

© 2023 Yang L et al. *JAMA Network Open*.
