# Supplementary material for: Comparison of Female Ovarian Reserve Before vs After COVID-19 Vaccination
Source: JAMA Netw Open. 2023 Jun 16;6(6):e2318804. doi: 10.1001/jamanetworkopen.2023.18804 (PMC10276301; doi:10.1001/jamanetworkopen.2023.18804)
Supplement: Supplement 2. — Data Sharing Statement [file jamanetwopen-e2318804-s002.pdf]

Data Sharing Statement

Yang. Comparison of Female Ovarian Reserve Before vs After COVID-19 Vaccination. *JAMA Netw Open*. Published June 16, 2023. doi:10.1001/jamanetworkopen.2023.18804

Data
Data available: No

Additional Information
Explanation for why data not available: Data will not be shared because sharing it has not been approved by the Institutional Review Board.
